# Acanthocytosis Contributes to the Diagnosis of the McLeod Syndrome

**DOI:** 10.1002/ajh.27755

**Published:** 2025-07-01

**Authors:** D. Mark Layton, Barbara J. Bain

**Affiliations:** ^1^ Centre for Haematology Hammersmith Hospital Campus of Imperial College London Faculty of Medicine London UK; ^2^ Centre for Haematology St Mary's Hospital Campus of Imperial College London Faculty of Medicine London UK

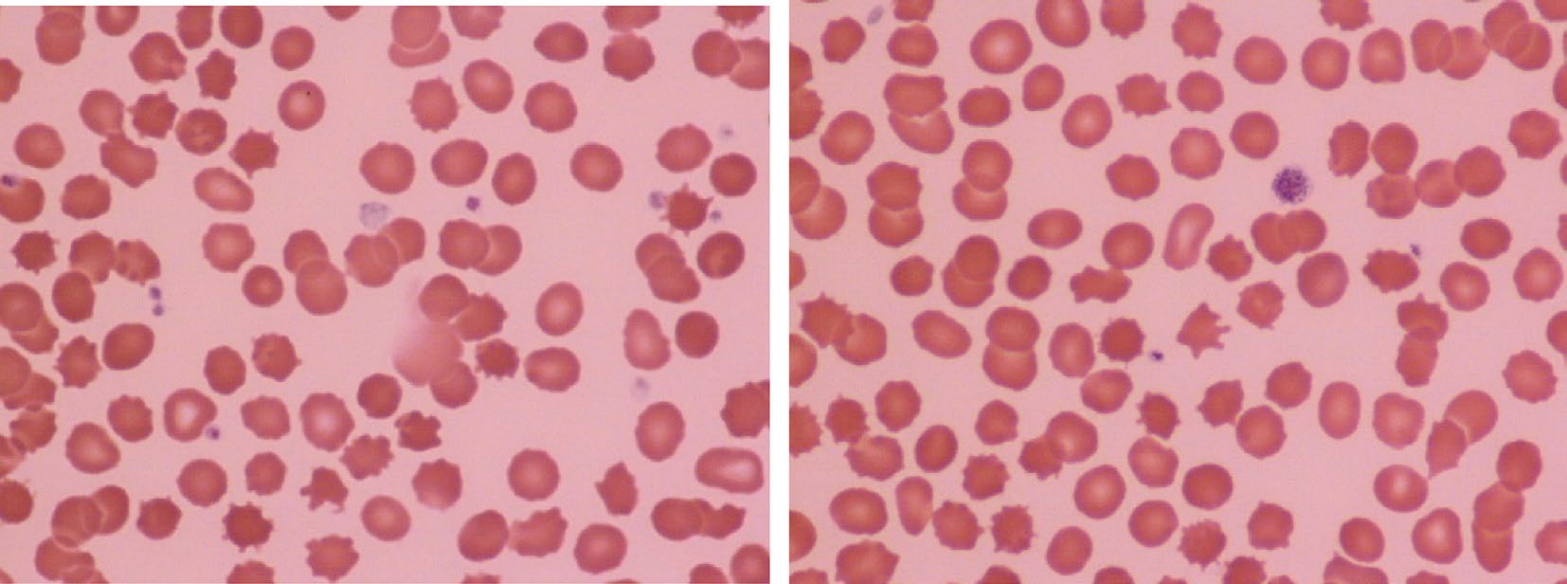

A 47‐year‐old man was referred to the hematology clinic after being found to have a low hemoglobin A1c level on routine screening. His fasting blood glucose was normal. He had a previous history of focal (temporal lobe) epilepsy first manifest in childhood with recent onset of generalized tonic clonic seizures and depression for which he had received treatment with carbamazepine, sodium valproate, zonisamide, clobazam, and citalopram. He reported mild cognitive impairment with memory loss and “brain fog.” Physical examination was unremarkable. A blood count showed hemoglobin concentration 141 g/L, mean cell volume 94.1 fL, mean cell hemoglobin 34.1 pg, mean cell hemoglobin concentration 362 g/L, and reticulocyte count of 152 × 10^9^/L. Bilirubin was normal but haptoglobin was reduced at 0.16 g/L (normal range 0.5–2.0) and lactate dehydrogenase raised at 254 U/L (125–243). A direct antiglobulin test was negative. On inspection a blood film showed numerous acanthocytes (images). The differential diagnosis thus included the McLeod syndrome and blood group typing showed the absence of Kell antigens. Genotyping identified a pathogenic variant of the *XK* gene c.268delT; p.(Tyr90fs) previously reported in two members of a family with neuroacanthocytosis due to the McLeod syndrome [1]. Further assessment following the diagnosis of McLeod syndrome revealed no evidence of a movement disorder or cardiomyopathy. Creatine kinase was raised at 540 U/L (40–320).

The McLeod syndrome results from mutation of the *KX* gene at Xp21.1. This gene encodes the KX protein to which, in the red cell membrane, Kell glycoprotein is bound. In addition to the typical red cell abnormality, the syndrome is characterized by late onset muscular and neurological defects including muscular wasting, cardiomyopathy, choreiform movements, epileptiform convulsions, psychiatric manifestations and cognitive impairment. There may also be mild anemia or compensated hemolysis. Observation of acanthocytosis can point to this diagnosis.

## Conflicts of Interest

The authors declare no conflicts of interest.
